# Astrocytic theory of working memory

**DOI:** 10.1186/1471-2202-15-S1-P206

**Published:** 2014-07-21

**Authors:** Maurizio De Pittà, Eshel Ben-Jacob, Hugues Berry

**Affiliations:** 1 EPI Beagle, INRIA Rhône-Alpes, Villeurbanne, France; 2 LIRIS, Université de Lyon, UMR5205 CNRS-INSA, Villeurbanne, France; 3 Department of Statistics, The University of Chicago, 5734 S. University Ave., Chicago, IL, USA; 4 School of Physics and Astronomy, Tel Aviv University, Ramat Aviv, Israel; 5 Center for Theoretical Biological Physics, Rice University, Houston, TX, USA

## 

**Figure 1:**
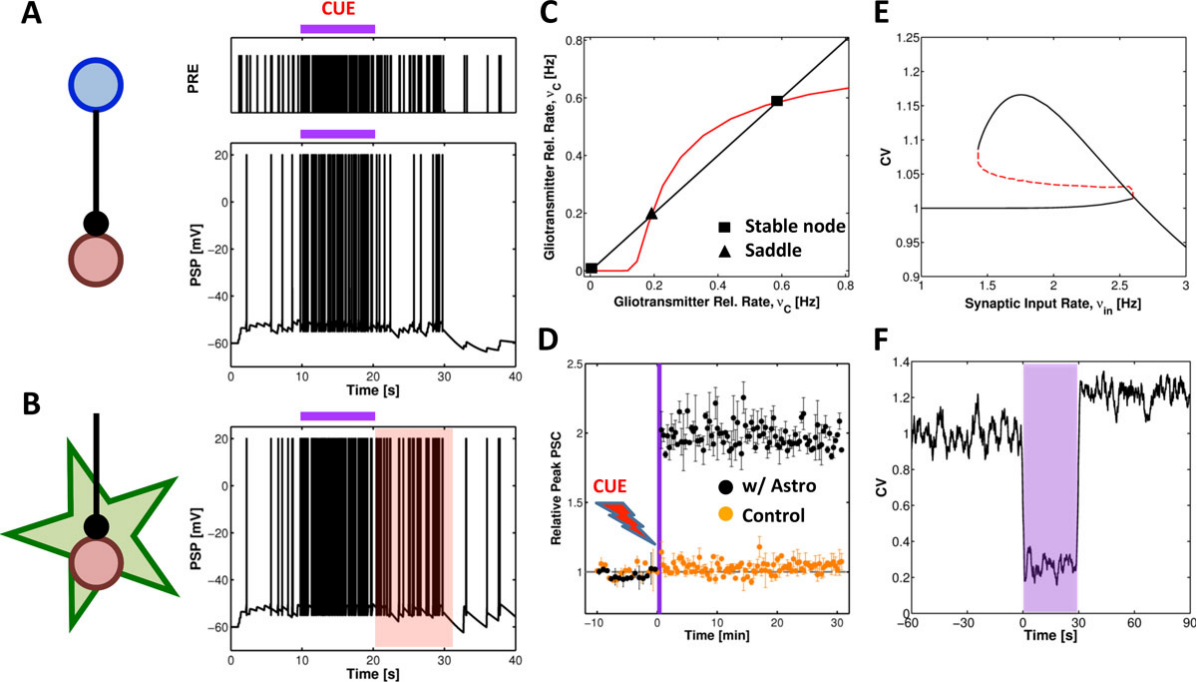
Astrocyte-based mechanism of working memory (WM). **A** Simulated firing activity of a cortical neuron before, during and after the presentation of a stimulatory cue (*purple bar*). **B** The same cue induces persistent firing activity in presence of astrocyte-mediated synaptic facilitation for more than 10 seconds (*red shade*). **C** Astrocyte-mediated facilitation results in bistability of synaptic release. **D** Synaptic release enhanced by the astrocyte may outlast the stimulatory cue as long as the astrocyte is kept active by the very synaptic stimuli impinging on the astrocyte. **E** The resulting neuronal firing statistics is more irregular in presence of the astrocyte, as marked by (**F**) a coefficient of variation (CV) larger than 1 after presentation of the cue (*purple shade*).

Working memory (WM) is the ability to transiently hold and manipulate goal-related information to forthcoming actions. In primates performing delayed-response tasks, the neural correlate of WM is the emergence of selective persistent activity in the prefrontal cortex (PFC), during the delay period – i.e., the increase of neuronal firing rates compared to baseline in response of specific stimulatory cues. Neuronal firing during such persistent activity can be highly irregular, with a coefficient of variation (CV) of the interspike interval distribution larger than one. The origin of this increased irregularity is a matter of debate, as it is not known whether it stems from inherent properties of neurons or the plasticity of their synaptic connections or both. Astrocytes, the main type of glial cells in the cortex, have recently emerged as potential active players in synaptic plasticity due to their proposed ability to regulate synaptic neurotransmitter release in response to neuronal activity. Because available WM models do not take into account astrocytes, we investigated the possible role of astrocyte regulation of synaptic plasticity in the emergence of persistent neuronal firing in WM and its statistics. Our modeling study suggests that selective persistent activity could indeed emerge from astrocyte-mediated short-term synaptic facilitation triggered by the brief presentation of a stimulatory cue (Figures 1A,B). Bifurcation analysis revealed that astrocyte-mediated facilitation could outlast the cue and switch to a self-sustained mode where it is maintained by the ongoing synaptic activity, consistent with the existence of bistability of synaptic release (Figures 1C,D). In parallel, mean field analysis showed that such bistable synaptic release resulted in bistability of neural firing rates, with persistent “UP” states characterized by larger irregularity (Figures 1E,F). Altogether, these results suggest a novel astrocyte-based mechanism for WM providing experimentally testable hypotheses for the possible involvement of astrocytes in WM-related cognitive tasks.

